# The Transcriptional Differences of Avian CD4^+^CD8^+^ Double-Positive T Cells and CD8^+^ T Cells From Peripheral Blood of ALV-J Infected Chickens Revealed by Smart-Seq2

**DOI:** 10.3389/fcimb.2021.747094

**Published:** 2021-11-10

**Authors:** Manman Dai, Li Zhao, Ziwei Li, Xiaobo Li, Bowen You, Sufang Zhu, Ming Liao

**Affiliations:** ^1^ National and Regional Joint Engineering Laboratory for Medicament of Zoonosis Prevention and Control, Guangdong Provincial Key Laboratory of Zoonosis Prevention and Control, College of Veterinary Medicine, South China Agricultural University, Guangzhou, China; ^2^ Core Facilities for Medical Science, Sun Yat-sen University, Guangzhou, China

**Keywords:** CD4^+^CD8^+^ double-positive T cell, CD8^+^ T cell, CD8^high^αα^+^ T cell, chicken, PBMCs, Smart-seq2

## Abstract

It is well known that chicken CD8^+^ T cell response is vital to clearing viral infections. However, the differences between T cell subsets expressing CD8 receptors in chicken peripheral blood mononuclear cells (PBMCs) have not been compared. Herein, we used Smart-Seq2 scRNA-seq technology to characterize the difference of chicken CD8^high+^, CD8^high^ αα^+^, CD8^high^ αβ^+^, CD8^medium+^, and CD4^+^CD8^low+^ T cell subsets from PBMCs of avian leukosis virus subgroup J (ALV-J)-infected chickens. Weighted gene co-expression network analysis (WGCNA) and Trend analysis revealed that genes enriched in the “Cytokine–cytokine receptor interaction” pathway were most highly expressed in the CD8^high^ αα^+^ T cell population, especially T cell activation or response-related genes including CD40LG, IL2RA, IL2RB, IL17A, IL1R1, TNFRSF25, and TNFRSF11, suggesting that CD8^high^ αα^+^ T cells rather than other CD8 subpopulations were more responsive to ALV-J infections. On the other hand, genes involved in the “FoxO signaling pathway” and “TGF-beta signaling pathway” were most highly expressed in the CD4^+^CD8^low+^ (CD8^low+^) T cell population and the function of CD4^+^CD8^low+^ T cells may play roles in negatively regulating the functions of T cells based on the high expression of CCND1, ROCK1, FOXO1, FOXO3, TNFRSF18, and TNFRSF21. The selected gene expressions in CD8^+^ T cells and CD4^+^CD8^low+^ double-positive T cells confirmed by qRT-PCR matched the Smart-Seq2 data, indicating the reliability of the smart-seq results. The high expressions of Granzyme K, Granzyme A, and CCL5 indicated the positive response of CD8^+^ T cells. Conversely, CD4^+^CD8^+^ T cells may have the suppressor activity based on the low expression of activation molecules but high expression of T cell activity suppressor genes. These findings verified the heterogeneity and transcriptional differences of T cells expressing CD8 receptors in chicken PBMCs.

## Introduction

Avian leukosis virus subgroup J (ALV‐J) can cause neoplastic disease, immunosuppression, and other production problems, resulting in huge economic losses for poultry industries. To date, there have been no vaccines or drugs available for the control of ALV-J infection. Our recent study found that CD8^+^ T cell responses were a potential key defense against ALV‐J infection (Dai et al., 2020). More interestingly, we found that a CD8^high+^ population increased in peripheral blood mononuclear cells (PBMCs) from ALV-J infected chickens compared to control chickens at 21 days post-infection (DPI), which then formed three stable populations of CD8^+^ T lymphocytes in the infected chickens, including CD8 ^high+^, CD8^medium+^, and CD4^+^CD8^low+^cells (Dai et al., 2020). Chicken CD4^+^CD8^low+^ T cells co-express CD4 and CD8alpha, but not CD8beta, and account for a large cell proportion in PBMCs (Luhtala et al., 1997), but its function was not understood. Conversely, CD8 ^high+^ and CD8^medium+^ T cells co-express CD8alpha and CD8beta, but not CD4, and occupy a small proportion in PBMCs (Dai et al., 2020). CD8^+^ T cells usually perform cytotoxic T cell functions after activation, but the difference of CD8^high+^ and CD8^medium+^ T cells in gene expression profiles was unknown. In addition, the differences of CD8αα and CD8αβ, the two phenotypes of CD8^+^ T cells, are not understood. The difference in avian CD4^+^CD8^+^ double-positive T cell and CD8^+^ T cell remains uninvestigated, which greatly limited our understanding of the heterogeneity and biology of chicken T cells.

Due to the limited numbers of CD8 ^high+^, CD8^high^ αα^+^, CD8^high^ αβ^+^, CD8^medium+^, and CD4^+^CD8^low+^ T cells in chicken PBMCs, we used single-cell RNA-seq (scRNA-seq) technology to analyze gene expression profiles after pathogen stimulation. Recent advances in scRNA-seq have provided approaches to investigate heterogeneous populations of T cells and have rapidly become a common tool for molecular profiling and identifying novel immune cell subsets and functions (Van Der Byl et al., 2019). The analyses of scRNA-seq data derived from plate-based sorted T cells using flow cytometry and full-length transcriptome protocols such as Smart-Seq2 scRNA-seq are very favorable for comparing the specific and low frequency of T cell populations. Moreover, to our knowledge, Smart-Seq2 scRNA-seq technology has never been applied in avian immunological research.

Herein, we performed Smart-Seq2 scRNA-seq technology to characterize the difference of chicken CD8^high+^, CD8^high^ αα^+^, CD8^high^ αβ^+^, CD8^medium+^, and CD4^+^CD8^low+^ T cells subsets derived from PBMCs of ALV-J infected chickens at 21 DPI, in order to identify the heterogeneity and potential function of T cells expressing CD8 receptors.

## Materials and Methods

### Ethics Statement

All animal trials were approved by the South China Agriculture University Institutional Animal Care and Use Committee (identification code: 2019076, 10 June 2019). All animal procedures were performed according to the regulations and guidelines established by this committee and international standards for animal welfare.

### Sample Preparation

PBMCs samples from three ALV-J infected chickens (#5, #12, and #15) at 21 DPI were prepared as previously described (Dai et al., 2020). In particular, when compared with uninfected chickens, a CD8^high+^ population increased in the ALV-J infected chicken PBMCs at 21 DPI, which formed three stable populations of CD8+ T lymphocytes, including CD8 ^high+^, CD8^medium+^, and CD4^+^CD8^low+^ T lymphocytes. CD4^+^CD8^low+^ T also named CD8^low+^ T cell in this study. The CD8^high+^ T lymphocyte included two phenotypes, CD8αα and CD8αβ (Dai et al., 2020). To compare and analyze the difference of these T lymphocytes populations, we subjected the sorted cell subsets to SMART-Seq2 based scRNA-seq analysis.

### Cell Sorting for SMART-Seq2 Based scRNA-Seq

A fluorescence-activated cell sorting machine (FACS Aria II, Becton Dickinson, New Jersey, USA) was used to sort a single cell into each well of a 96-well PCR plate containing 2.5μL of 10× Lysis Buffer (Vazyme# N711). Pooled PBMCs were from the mixed equal amount of PBMC samples of three ALV-J infected chickens at 21 DPI. For the isolation of CD8^high+^, CD8^medium+^, and CD4^+^CD8^low+^ (CD8^low+^) populations, the pooled PBMCs from ALV-J infected chicken were stained for APC‐conjugated mouse anti‐chicken CD3^+^, FITC‐conjugated mouse anti‐chicken CD4^+^, and PE‐conjugated mouse anti‐chicken CD8α^+^ monoclonal antibodies (Southern Biotech, Birmingham, USA). For CD8^high^ αα^+^ and CD8^high^ αβ^+^ population isolation, the pooled PBMCs from ALV-J infected chicken were stained for APC‐conjugated mouse anti‐chicken CD3^+^, PE‐conjugated mouse anti‐chicken CD8α^+,^ and FITC‐conjugated mouse anti‐chicken CD8β^+^ monoclonal antibodies (Southern Biotech, Birmingham, USA) as described previously (Dai et al., 2020). Each population of five T lymphocyte subtypes was analyzed in four replications, and each replication sorted 100 single cells for subsequent SMART-Seq2 analysis. Furthermore, the gating strategy for each population was shown in **Supplementary Figures 1A, B**.

### Library Preparation for SMART-Seq2 Based scRNA-Seq

Library construction and sequencing were completed by Gene Denovo (Guangzhou, China). Total RNA from the lysed cells was briefly released, enriched by Oligo (dT) primers, and reverse transcribed into cDNA using the Discover-sc WTA Kit V2 (Vazyme). cDNA was purified with VAHTS DNA Clean Beads. Sequencing libraries were constructed according to the TruePrepTM DNA Library Prep Kit V2 (Vazyme #TD503). Lastly, library quality was assessed on an Agilent 2100 Bioanalyzer (Agilent Technologies, Palo Alto, CA, USA) and sequenced using Illumina Novaseq6000 by Gene Denovo Biotechnology Co. (Guangzhou, China).

### SMART-Seq Sequencing Data Processing and Quality Control

Reads obtained from the sequencing machines were further filtered using fastp (Chen et al., 2018) (version 0.18.0). The parameters were as follows: 1) removal of reads containing adapters; 2) removal of reads containing more than 10% of unknown nucleotides (N); 3) removal of low-quality reads containing more than 50% of low-quality bases (Q-value ≤20). Furthermore, the ribosome RNA (rRNA) mapped reads were removed *via* the short reads alignment tool Bowtie2 (Langmead and Salzberg, 2012) (version 2.2.8). The remaining clean reads were mapped to the chicken genome of GRCg6a (Zerbino et al., 2018) using HISAT2.2.4 (Kim et al., 2015). FPKM (fragment per kilobase of transcript per million mapped reads) value was calculated to quantify the gene expression abundance using RSEM (Li and Dewey, 2011) software. Principal component analysis (PCA) was performed with the R package gmodels (http://www.rproject.org/) to reveal various samples’ repeatability and potential relationship.

### Differentially Expressed Genes

Differential expression RNA analysis was performed using DESeq2 (Love et al., 2014) software comparing two different groups. Differentially expressed genes (DEGs) were identified with the parameter of false discovery rate (FDR) <0.05 and absolute fold change (FC) ≥2. The Kyoto Encyclopedia of Genes and Genomes (KEGG) (Kanehisa and Goto, 2000), an important public pathway-related database, was used for pathway enrichment analysis. The analysis identified significantly enriched metabolic pathways or signal transduction pathways in DEGs compared with the whole genome background with a false discovery rate threshold (FDR) < 0.05.

### Weighted Gene Co-Expression Network Analysis

Weighted gene co-expression network analysis (WGCNA) is a system biology method used to describe the correlation patterns among genes across multiple samples and identify modules of highly correlated genes. After filtering 9862 genes with low expression in all samples, WGCNA (v1.47) package in R (Langfelder and Horvath, 2008) was used to construct co-expression modules based on the gene expression values. Module Eigengenes were used to calculate the correlation matrices with samples to identify biologically significant modules. Intramodular connectivity of each gene was calculated, and high connectivity tended to be hub genes with potentially important functions. The networks were visualized using Cytoscape (v3.7.1). For genes in each module, KEGG pathway enrichment analysis was conducted to analyze the biological functions of each module.

### Trend Analysis

Gene expression pattern analysis was used to cluster genes sharing similar expression patterns for multiple samples. The expression data of each sample were normalized to 0, log2 (v1/v0), log2 (v2/v0), and then clustered by Short Time-series Expression Miner software (STEM, version 1.3.11) to examine the expression pattern of DEGs (Ernst and Bar-Joseph, 2006). The parameters were set as follows: 1) maximum unit change in model profiles between time points was 1; 2) maximum output profiles number was 20, and similar profiles were merged; 3) a minimum ratio of FC of DEGs was no less than 2.0. The clustered profiles with p-values ≤0.05 were considered as significant profiles. Then the DEGs in each profile were subjected to KEGG pathway enrichment analysis.

### Protein-Protein Interaction

A protein-protein interaction (PPI) network was first identified based on WGCNA analysis as described above. Next, the interaction network was supplemented with String v10 (Szklarczyk et al., 2015), which determined genes as nodes and interactions as lines in a network. The network file was visualized using Cytoscape (v3.7.1) (Shannon et al., 2003) software to present a core and hub gene biological interaction.

### Real-Time PCR Confirmation of the SMART-Seq2 Data

Primers for quantitative reverse transcription PCR (qRT-PCR) were designed using the National Center for Biotechnology Information (NCBI) Primer-BLAST program. The GAPDH gene was used as an internal control. All primers used in this study are listed in **Supplementary File 1**. A total of 2×10^5^ live cells of CD8^+^ T cell or CD4^+^CD8^low+^ double-positive T cell from three ALV-J infected chickens (#5, #12, and #15) at 21 DPI were respectively sorted for total RNA extraction. Furthermore, the gating strategy for each population is shown in **Supplementary Figure 1C**. qRT-PCR was performed on an ABI7500 Real-Time PCR system (Applied Biosystems, USA) using ChamQ Universal SYBR qPCR Master Mix (Vazyme, Nanjing, China) according to the manufacturer’s specifications. Expression levels of the tested reference genes were determined by CT values and calculated by 2^-△△Ct^ (Livak and Schmittgen, 2001). Data were collected from three biological samples in each group, and each sample was performed in triplicate.

### Statistical Analysis

Statistical comparisons were made by GraphPad Prism 8 (GraphPad Software Inc., San Diego, CA, USA). The results were presented as mean ± SEM. The paired or unpaired t-test was used for statistical comparison. *P < 0.05, **P < 0.01, ***P < 0.001.

## Results

### WGCNA Analysis of CD4^+^CD8^low+^, CD8^medium+^, CD8^high+^, CD8^high^ αα^+^ and CD8^high^ αβ^+^ T Cell Populations in PBMCs From ALV-J Infected Chicken at 21 DPI

**Figure 1:**
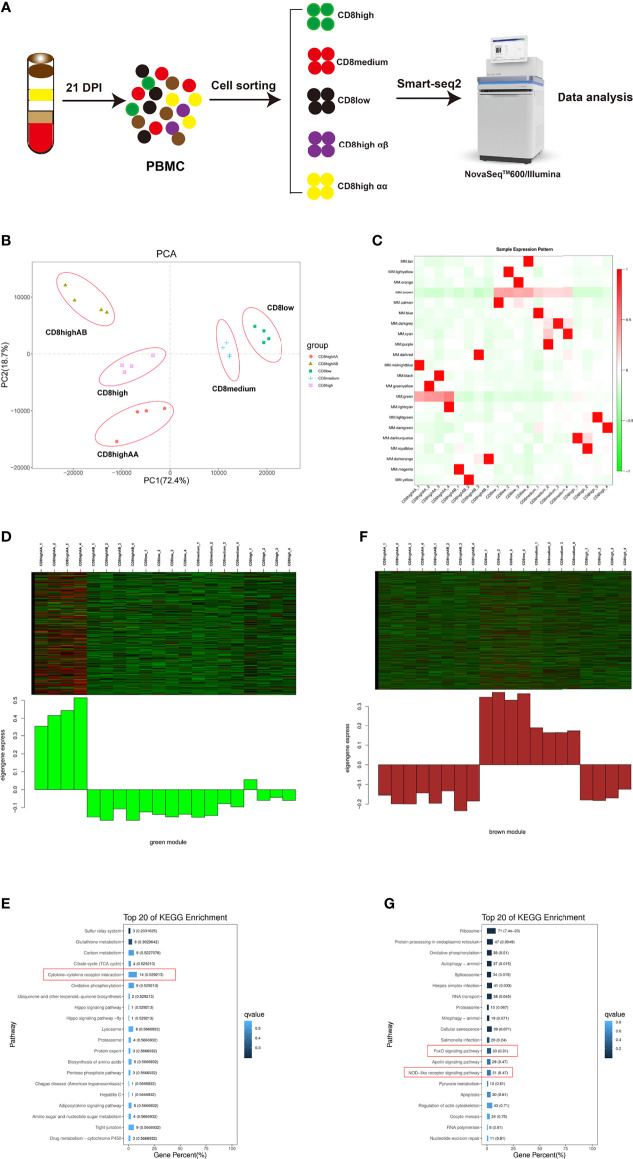
WGCNA analysis of CD4^+^CD8^low+^ (CD8^low+^), CD8^medium+^, CD8^high+^, CD8^high^ αα^+^, and CD8^high^ αβ^+^ T cell populations in PBMCs from ALV-J infected chicken at 21 days post-infection. **(A)** Schematic outline of the study design. **(B)** PCA analysis of the five T cell populations, each with four replications. The X- and Y-axis show the principal component 1 and component 2, which explains 72.4% and 18.7% of the total variance. **(C)** The heat map of sample expression pattern. Red indicates high expression. Green indicates low expression. **(D)** Heat map of the gene expression pattern of the green module. **(E)** Top 20 KEGG pathways selected for green module gene enrichment. **(F)** Heat map of the gene expression pattern of the brown module. **(G)** Top 20 KEGG pathways selected for brown module gene enrichment.

We used the fluorescent cell sorting based SMART-Seq2 platforms to perform full-length scRNA-seq on five T lymphocytes populations including CD4^+^CD8^low+^, CD8^medium+^, CD8^high+^, CD8^high^ αα^+^, and CD8^high^ αβ^+^ T cells in PBMCs from ALV-J infected chicken at 21 DPI (**Figure 1A** and **Supplementary Figures 1A, B**). Each T cell population obtained in four replicate experiments showed excellent repeatability based on the PCA and Pearson’s correlation analysis (**Figure 1B** and **Supplementary Figure 2**). Then we explored the correlation patterns among genes across the twenty samples *via* WGCNA analysis. Genes were clustered into 22 correlated modules (**Figure 1C** and **Supplementary File 2**). Moreover, we found that CD8^high^ αα^+^ T cells were correlated with the green module in which genes were enriched in the immune pathway “Cytokine–cytokine receptor interaction” (**Figures 1D, E** and **Supplementary File 3**), while CD4^+^CD8^low+^ and CD8^medium+^ T cells were related to the brown module in which genes were enriched in immune pathways “FoxO signaling pathway” and “NOD-like receptor signaling pathway” (**Figures 1F, G** and **Supplementary File 4**).

### Trend Analysis of CD8^high+^, CD8^high^ αα^+^, and CD8^high^ αβ^+^ T Cells

**Figure 2:**
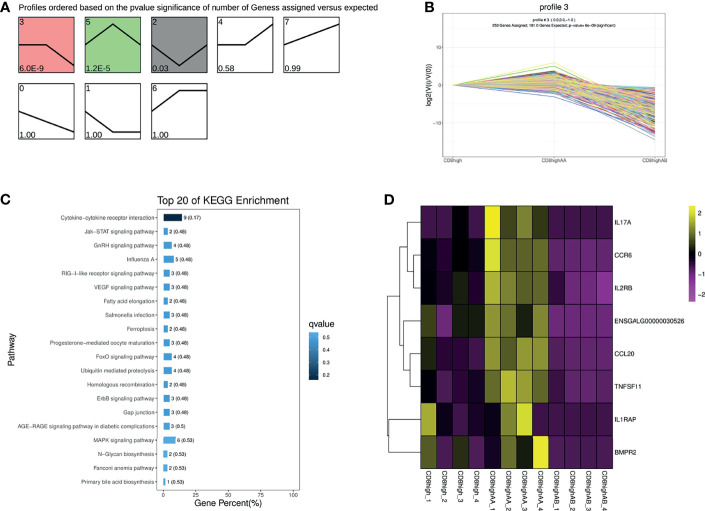
Trend analysis of CD8^high+^, CD8^high^ αα^+^, and CD8^high^ αβ^+^ T cells. **(A)** All differentially expressed gene (DEG) expression profiles ordered based on the p values in the order of CD8^high+^ T cells (containing CD8^high^ αα^+^ and CD8^high^ αβ^+^ T cells), CD8^high^ αα^+^ T cells and CD8^high^ αβ^+^ T cells. **(B)** The DEG expression trends in profile 3. **(C)** Top 20 KEGG pathways were selected for profile 3 DEGs enrichment. **(D)** Heat map of DEGs expression enriched in the “Cytokine–cytokine receptor interaction” pathway.

To further investigate the heterogeneous populations of the five chicken T lymphocytes populations, we first performed the trend analysis of CD8^high+^, CD8^high^ αα^+,^and CD8^high^ αβ^+^ T cells. CD8^high+^ T cell contained both CD8^high^ αα^+^ and CD8^high^ αβ^+^ T cells. The most significant profile, profile 3, displayed a slightly increased then decreased trend in the order of CD8^high+^ T cell, CD8^high^ αα^+^ T cell, and CD8^high^ αβ^+^ T cell (**Figures 2A, B**). Next, 253 genes in profile 3 were subjected to KEGG enrichment analysis, in which genes were mainly enriched in the “Cytokine–cytokine receptor interaction” pathway (**Figure 2C**, **Supplementary File 5**). The heat map results showed that genes enriched in the “Cytokine–cytokine receptor interaction” pathway were highly expressed in the CD8^high^ αα^+^ T cell population (**Figure 2D** and **Supplementary File 5**).

### Trend Analysis of CD4^+^CD8^low+^, CD8^medium+^, and CD8^high+^ T Cells

**Figure 3:**
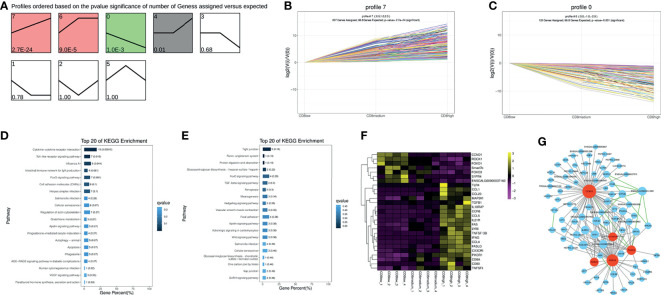
Trend analysis of CD4^+^CD8^low+^, CD8^medium+^ and CD8^high+^ T cells. **(A)** All differentially expressed genes (DEGs) expression profiles were ordered based on the *P*-values of CD4^+^CD8^low+^, CD8^medium+,^ and CD8^high+^ T cells. **(B)** The DEGs expression trends in profile 7. **(C)** The DEGs expression trends in profile 0. **(D)** Top 20 KEGG pathways were selected for profile 7 DEGs enrichment. **(E)** Top 20 KEGG pathways were selected for profile 0 DEGs enrichment. **(F)** Heat map of picked-up immune genes. **(G)** Gene interaction network analysis based on the WGCNA analysis and STRING database. The gray line indicates the interaction relationship based on the WGCNA analysis. The green line indicates the potential interaction based on the STRING database. Red nodes represent hub genes.

Similarly, trend analysis for CD4^+^CD8^low+^, CD8^medium+^, and CD8^high+^ T cells was performed. Profile 7 displayed a significantly up-regulated pattern, and profile 0 showed a down-regulated pattern in the order of CD4^+^CD8^low+^, CD8^medium+^ and CD8^high+^ T cells (**Figures 3A**–**C**). In addition, genes in profile 7 were mainly enriched in the “Cytokine–cytokine receptor interaction” and “Toll-like receptor signaling pathway” and were highly expressed in the CD8^high+^ T cells population as could be expected (**Figures 3D, F** and **Supplementary File 6**). For example, immune-related genes including IL18RAP, CCR6, CCL4, CCL5, FAS, and IFNG were predominantly expressed in the CD8 ^high+^ T cells population (**Figure 3F**). Genes in profile 0 were mainly involved in the “FoxO signaling pathway” and “TGF-beta signaling pathway” and were highly expressed in the CD4^+^CD8^low+^ (CD8^low+^) T cells population (**Figures 3E, F** and **Supplementary File 6**).

The seven predominantly expressed genes in the CD8^low+^ T cells population include CCND1, ROCK1, FOXO1, Smad7b, FOXO3, S1PR4, and ENSGALG00000037160, and we also conducted an interaction network analysis based on WGCNA analysis and STRING database. The results implied that CCND1, ROCK1, FOXO1, FOXO3, and ENSGALG00000037160 were the important hub genes (**Figure 3G**, marked in red), and their regulation on chicken T cells functions had not been reported. In mammals, it has been reported that FOXO1 or FOXO3 plays an essential role in specifying the program of T cell differentiation, most importantly in the pathway leading to the development and function of regulatory T (Treg) cells (Kerdiles et al., 2010; Ouyang et al., 2012). Further, CCND1 has been reported to be negatively correlated with T cell activation (Lu et al., 2019). ROCK was also the key regulator of T-lymphocyte development, activation, and differentiation (Saoudi et al., 2014), and the RhoA-ROCK pathway is the specific pathway for Th1, Th17, and Treg responses (Liu et al., 2020). This information suggests that the function of CD4^+^CD8^low+^ T cells may be involved in negatively regulating the activity of T cells, based on the high expression of CCND1, ROCK1, FOXO1, and FOXO3.

### Gene Expression Analysis Enriched in the “Cytokine–Cytokine Receptor Interaction” Pathway of CD4^+^CD8^low+^, CD8^medium+^, CD8^high+^, CD8^high^ αα^+^, and CD8^high^ αβ^+^ T Cell Populations

**Figure 4:**
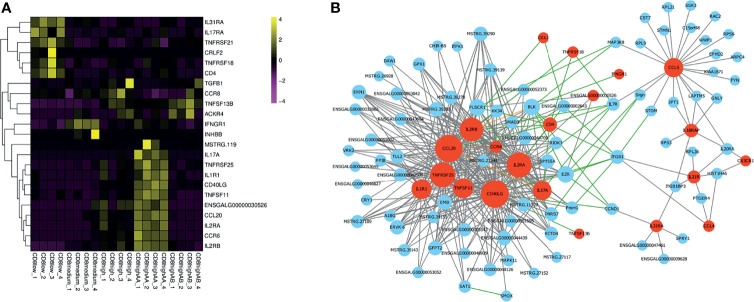
Gene expression analysis enriched in the “Cytokine–cytokine receptor interaction” pathway of CD4^+^CD8^low+^, CD8^medium+^, CD8^high+^, CD8^high^ αα^+,^and CD8^high^ αβ^+^ T cell populations. **(A)** Heat map of selected immune genes enriched in the “Cytokine–cytokine receptor interaction” pathway. **(B)** Gene interaction network analysis based on WGCNA analysis and STRING database. The gray line indicates the potential interaction relationship based on the WGCNA analysis. The green line indicates the potential interaction relationship based on the STRING database. The pink line indicates the potential interaction based on both the WGCNA analysis and STRING database. Red nodes represent hub genes.

To further compare the gene expression characteristics of CD4^+^CD8^low+^, CD8^medium+^, CD8^high+^, CD8^high^ αα^+,^and CD8^high^ αβ^+^ T cell populations, a total of 23 significant genes enriched in the “Cytokine–cytokine receptor interaction” pathway were screened, and their expression levels were quantified in a heat map (**Figure 4A** and **Supplementary File 7**). We also performed an interaction network analysis of these genes based on the WGCNA analysis and the STRING database (**Figure 4B**). The results showed that the hub genes including IL17A, CD40LG, IL2RA, IL2RB, CCR6, CCL20, TNFRSF25, TNFRSF11, and IL1R1 were predominantly expressed in CD8^high^ αα^+^ T cells (**Figure 4**). Conversely, hub genes including CD4, IL31RA, and TNFRSF18 were highly expressed in CD4^+^CD8^low+^ T cells (**Figure 4**).

### Analysis of the Selected Gene Expressions in CD8^+^ T Cell Compared to CD4^+^CD8^low+^ Double-Positive T Cell by Smart-Seq and qRT-PCR

**Figure 5:**
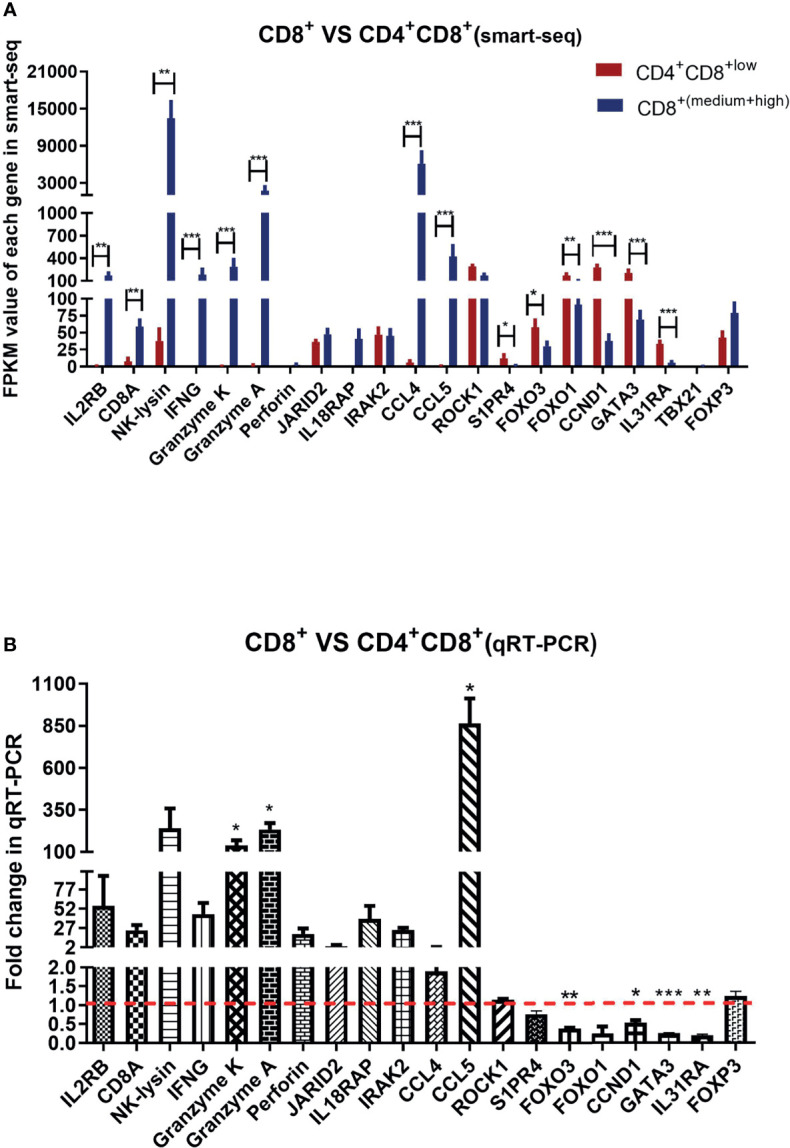
Analysis of the selected gene expressions in CD8^+^ T cell compared to CD4^+^CD8^low+^ double-positive T cell by smart-seq and qRT-PCR. **(A)** Analysis expression of randomly selected 21 DEGs in CD4^+^CD8^low+^ T cells and CD8^+^ T cells (containing CD8^medium+^ and CD8 ^high+^ T cells) of ALV-J infected chickens by smart-seq. The data shown are mean ± SE (n=4 in CD4^+^CD8^low+^ T cells, and n=8 in CD8^+^ T cells). **(B)** Analysis expression of 20 DEGs in CD8^+^ T cells compared to CD4^+^CD8^+^ T cells by qRT-PCR. Total RNA of sorted CD8^+^ T cells and CD4^+^CD8^low+^ double-positive T cells were extracted from three ALV-J infected chickens. The data was collected from three biological samples, and each sample was tested in triplicate. The results are presented as means ± SEM. Statistical comparisons were performed using GraphPad Prism. Statistical significance was assessed at *P*-values *P < 0.05, **P < 0.01, ***P < 0.001.

Given the limited cell numbers of CD8^high+^, CD8^high^ αα^+^ and CD8^high^ αβ^+^ T cells in PBMCs, we could not obtain sufficient cell numbers to perform qRT-PCR detection. Thus, we sorted the CD4^+^CD8^low+^ double-positive T cells and CD8^+^ T cells containing mixed CD8^medium+^ and CD8^high+^ T cell populations for qRT-PCR. The sorting gate strategy is shown in **Supplementary Figure 1C**. CD4^+^CD8^low+^ double-positive T cells and CD8^+^ T cells from three ALV-J infected chickens were sorted respectively to evaluate the reliability of the smart-seq results, and 20 DEGs were selected to validate the relative expression levels in the CD8^+^ T cells compared to CD4^+^CD8 ^low+^ T cells using qPCR. As shown in **Figures 5A, B**, the trends in expression of these randomly selected DEGs were consistent between smart-seq data and qRT-PCR data. Specifically, FOXO3, FOXO1, CCND1, GATA3, and IL31RA had a low expression, IL2RB, CD8A, NK-lysin, IFNG, Granzyme K, Granzyme A, and CCL5 were highly expressed in CD8^+^ T cells compared to CD4^+^CD8^low+^ double-positive T cells (**Figures 5A, B** and **Supplementary File 8**).

## Discussion

Previously, we found that the CD8^+^ T cell response was a potential key factor defending against ALV‐J infection, and a marked increase in the percentage of CD8^+^ T cells was detected at 21 DPI in the ALV-J infection group compared with the control group (Dai et al., 2020). However, the differences of various T cells subtypes were unknown. Herein, we exploit Smart-Seq2 scRNA-seq technology to evaluate the gene expression profiles of CD8^high+^, CD8^high^ αα^+^, CD8^high^ αβ^+^, CD8^medium+^, and CD4^+^CD8^low+^ T cells in PBMCs from ALV-J infected chickens at 21 DPI. Given that the majority of the CD8^high+^ populations were of the CD8αβ phenotype, the CD8^high^ αβ^+^ T cells was assumed to be more important for clearing viruses in our previous study (Dai et al., 2020). Conversely, in this study, we found that genes enriched in the immune pathway “Cytokine–cytokine receptor interaction” were highly expressed in CD8^high^ αα^+^ T cells, suggesting that CD8^high^ αα^+^ T cells instead of other CD8^+^ subpopulations were more effective in response to ALV-J infection. Besides, CD8^+^ γδ T cell also expressed CD8α co-receptor (Laursen et al., 2018), but we could not distinguish the CD8^+^ γδ T cell from the CD8α^+^ T cells in this study. Meanwhile, genes involved in the “FoxO signaling pathway” and the “TGF-beta signaling pathway” were highly expressed in the CD4^+^CD8^low+^ (CD8^low+^) T cell population, which implied that the function of CD4^+^CD8^low+^ T cells differed from CD8^+^ T cells. Specifically, highly expressed genes in CD4^+^CD8^low+^ T cells, including ROCK1 (Liu et al., 2020), FOXO1 (Ouyang et al., 2012), and FOXO3 (Kerdiles et al., 2010), have been reported to involve in the development and function of Treg, which suggests that the function of CD4^+^CD8^low+^ T cells may be involved in negative regulation of T cells functions.

The expression profile of immune-related genes also revealed that the T cell activation or response-related genes including CD40LG (Pasqual et al., 2018), IL2RA, IL2RB, IL17A (Bhat et al., 2017), IL1R1 (Sarkar et al., 2018), TNFRSF25 (Slebioda et al., 2011), and TNFRSF11 (Bishop et al., 2015) were mainly expressed in CD8^high^ αα^+^ T cells (**Figure 4**), which suggests that the ratio and number of CD8^high^ αα^+^ T cells could represent a potential marker of CD8^+^ T cell response. Conversely, highly expressed genes of CD4^+^CD8^low+^ T cells, including TNFRSF18, CD4, IL31RA, and TNFRSF21, implies its function differs from that of CD8^+^ T cells (**Figure 4**). For example, TNFRSF18 (also known as GITR) has been reported to augment the suppressive activity of Tregs (Igarashi et al., 2008; Azuma, 2019). TNFRSF21 (also known as DR6) is potentially involved in the negative regulation of the proliferation or function of CD4^+^ T cells (Liu et al., 2001). IL31RA, the receptor for the IL31 cytokine, is preferentially produced by T helper type 2 cells (Dillon et al., 2004). This information further suggests that the function of CD4^+^CD8^+^ T cells is more similar to that of CD4^+^ T cells than CD8^+^ T cells and is likely approximated to perform the suppressor activity.

Given the limited cell numbers of each CD8^+^ T cell population that can be isolated from chicken PBMCs, we sorted the CD4^+^CD8^low+^ double-positive T cells and mixed CD8^+^ T cells from ALV-J infected chickens to perform qRT-PCR. The selected gene expression trends for CD8^+^ T cells compared to CD4^+^CD8^low+^ double-positive T cell matched the Smart-Seq2 scRNA-seq data (**Figures 5A, B**), indicating that the scRNA-seq data were reliable. Specifically, cytotoxicity-associated genes, including Granzyme K and Granzyme A, and chemokine CCL5, were highly expressed in CD8^+^ T cells instead of CD4^+^CD8^low+^ double-positive T cells (**Figures 5A, B**). CCL5 was reported to mediate T cell chemotaxis (Murooka et al., 2008). Thus, the high expression of these genes indicated the positive response of CD8^+^ T cells to ALV-J infection.

Conversely, FOXO3 and CCND1 were lowly expressed in the CD8^+^ T cells but highly expressed in the CD4^+^CD8^low+^ double-positive T cells (**Figures 5A, B**). FOXO3 (Kerdiles et al., 2010) has been reported to involve in the development and function of Treg, and CCND1 is negatively correlated with T cell activation (Lu et al., 2019). The relatively high expressions of FOXO3 and CCND1 were indicative of the suppressor activity of CD4^+^CD8^low+^ T cells. Besides, we also detected the expressions of T help cell differentiation-related transcription factors including TBX21 (T-bet), GATA3, and Foxp3 between CD4^+^CD8^low+^ double-positive T cells and CD8^+^ single-positive T cells after ALV-J infection. Furthermore, no expression of TBX21 (related to Th1 polarization) was detected in both T cells, and the GATA3 was highly expressed in CD4^+^CD8^low+^ double-positive T cells compared to CD8^+^ single-positive T cells (**Figure 5**). GATA3 was the marker gene of Th2-polarized (Lone et al., 2021). In addition, we detected high expression of IL31RA, the receptor for the Th2 cytokine (IL31) (Dillon et al., 2004), in CD4^+^CD8^low+^ double-positive T cells. Therefore, we suspected that the function of CD4^+^CD8^+^ T cells was more similar to that of CD4^+^ T cells and could potentially differentiate into Th2 cells, which needs further verification in the future. Although no expression difference of Foxp3 (Treg-related transcription factor) was detected, we detected the low expression of effector molecules but high expression of T cell activity suppressor genes in the CD4^+^CD8^low+^ double-positive T cells (**Figures 3**–**5**). Accordingly, CD4^+^CD8^+^ T cells may be involved in negatively regulating the activity of T cells combined with the qPCR and smart-seq results.

In summary, we exploited Smart-Seq2 scRNA-seq technology to analyze the gene expression profiles of different T cell populations involving the CD8 receptor in PBMCs isolated from ALV-J infected chickens. We determined that the increase in CD8^high^ αα^+^ T cells represents an effective response to viral infection. The function of CD4^+^CD8^+^ double-positive T cells seems to be closer to that of CD4^+^ T cells than CD8^+^ T cells, and whose function is likely approximated to regulate the activity of T cells negatively. CD8^+^ T cells show positive responses to viral infection.

## Data Availability Statement

The datasets presented in this study can be found in online repositories. Sequencing data have been deposited in BioProject under the accession number PRJNA713900.

## Ethics Statement

All animal research projects were approved by the South China Agriculture University Institutional Animal Care and Use Committee (identification code: 2019076, 10 June 2019).

## Author Contributions

MD designed the study, performed experiments, collected and analyzed data, and drafted the manuscript. LZ, ZL, XL, BY, and SZ assisted with data analysis, cell sorting, and qRT-PCR. ML coordinated the study and revised the manuscript. All authors contributed to the article and approved the submitted version.

## Funding

This work was supported by the National Natural Science Foundation of China (32172868, 31802174, and 31830097), Young Elite Scientists Sponsorship Program by CAST (2020QNRC001), 111 Project (D20008).

## Conflict of Interest

The authors declare that the research was conducted in the absence of any commercial or financial relationships that could be construed as a potential conflict of interest.

## Publisher’s Note

All claims expressed in this article are solely those of the authors and do not necessarily represent those of their affiliated organizations, or those of the publisher, the editors and the reviewers. Any product that may be evaluated in this article, or claim that may be made by its manufacturer, is not guaranteed or endorsed by the publisher.

## Supplementary Material

Supplementary Figure 1 The sorting gate strategy of each cell population for single-cell RNA-seq (scRNA-seq) or real-time PCR. **(A)** Sorting gate strategy of CD8^low+^, CD8^medium+^ and CD8^high+^ T cells for SMART-Seq2 based scRNA-seq. **(B)** Sorting gate strategy of CD8^high^αα+ and CD8^high^αβ+ T cells for SMART-Seq2 based scRNA-seq. **(C)** Sorting gate strategy of CD4^+^CD8^low+^ and CD8^+^ T cells for Real-time PCR

Supplementary Figure 2 Pearson correlation coefficient between every two samples.

Supplementary File 1 The primers for quantitative reverse transcription PCR.

Supplementary File 2 Module eigenvalues of the module genes in each sample.

Supplementary File 3 Pathways for green module gene enrichment.

Supplementary File 4 Pathways for brown module gene enrichment.

Supplementary File 5 Pathways for profile 3 gene enrichment and DEGs expression analysis enriched in the “Cytokine–cytokine receptor interaction” pathway.

Supplementary File 6 Pathways for profile 0 or profile 7 gene enrichment, and picked up immune genes expression analysis.

Supplementary File 7 Selected immune genes expression analysis enriched in the “Cytokine–cytokine receptor interaction” pathway.

Supplementary File 8 Orignal data for **Figures 5A, B**.
