# Supplementary figures and images for: The Transcriptional Differences of Avian CD4+CD8+ Double-Positive T Cells and CD8+ T Cells From Peripheral Blood of ALV-J Infected Chickens Revealed by Smart-Seq2
Source: Front Cell Infect Microbiol. 2021 Nov 10;11:747094. doi: 10.3389/fcimb.2021.747094 (PMC8631335; doi:10.3389/fcimb.2021.747094)

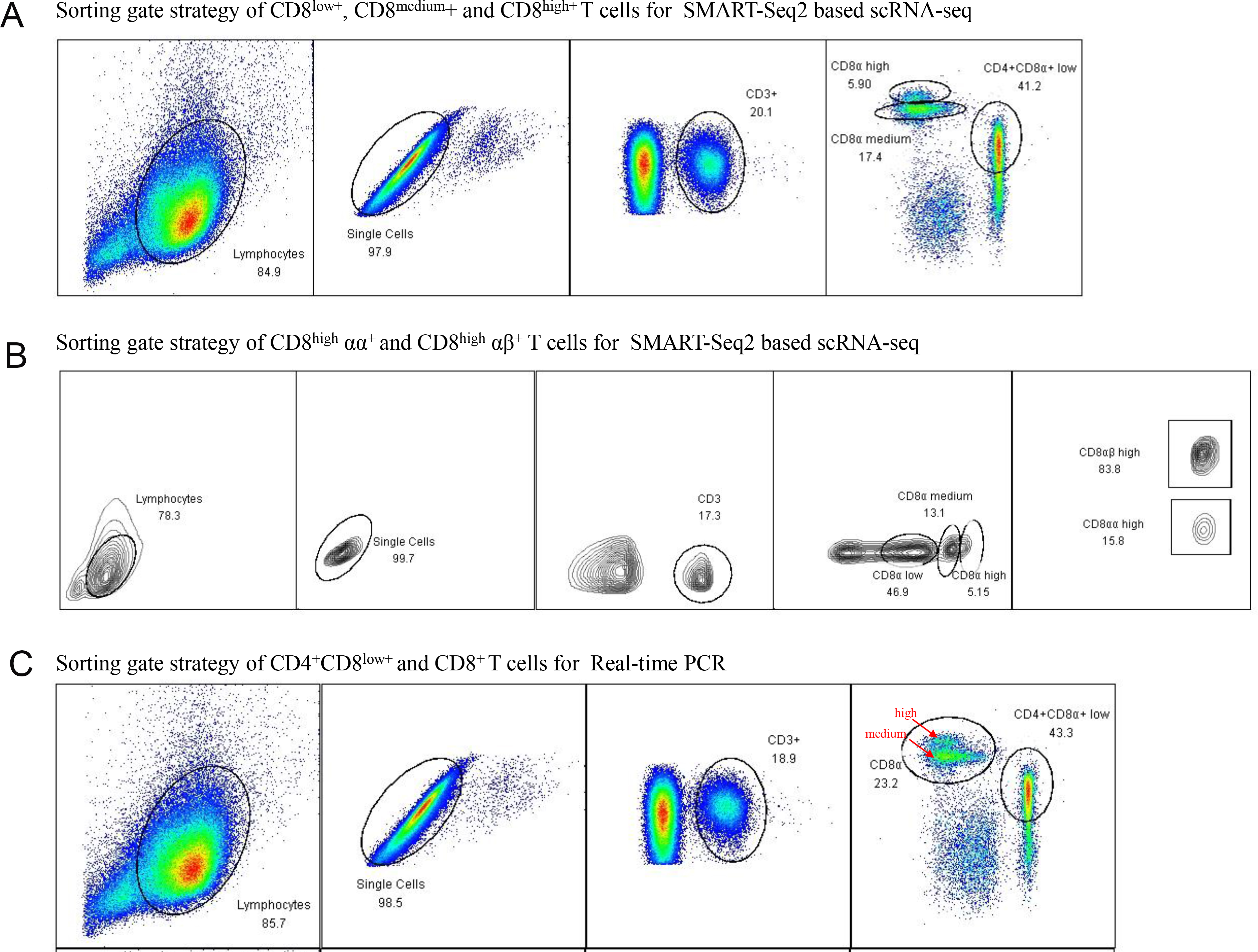

Supplement: Supplementary Figure 1 — The sorting gate strategy of each cell population for single-cell RNA-seq (scRNA-seq) or real-time PCR. (A) Sorting gate strategy of CD8low+, CD8medium+ and CD8high+ T cells for SMART-Seq2 based scRNA-seq. (B) Sorting gate strategy of CD8highαα+ and CD8highαβ+ T cells for SMART-Seq2 based scRNA-seq. (C) Sorting gate strategy of CD4+CD8low+ and CD8+ T cells for Real-time PCR [file Image_1.jpeg]

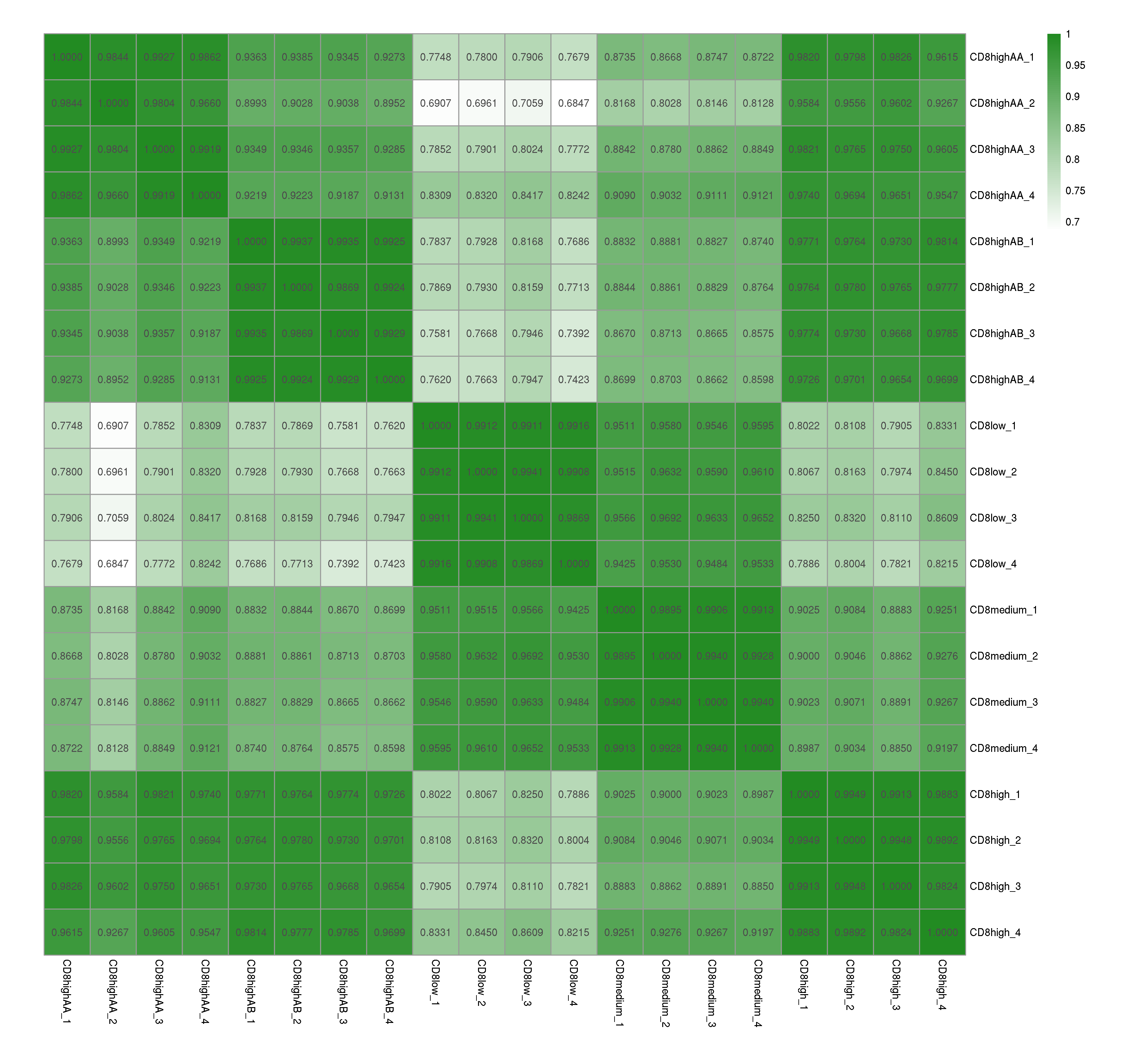

Supplement: Supplementary Figure 2 — Pearson correlation coefficient between every two samples. [file Image_2.png]
