# Supplementary material for: The Transcriptional Differences of Avian CD4+CD8+ Double-Positive T Cells and CD8+ T Cells From Peripheral Blood of ALV-J Infected Chickens Revealed by Smart-Seq2
Source: Front Cell Infect Microbiol. 2021 Nov 10;11:747094. doi: 10.3389/fcimb.2021.747094 (PMC8631335; doi:10.3389/fcimb.2021.747094)
Supplement: Supplementary File 1 — The primers for quantitative reverse transcription PCR. [file Table_1.docx]

Primers for qRT-PCR

| **Target** | **Primer** | **Sequence (5’~3’)** | **Gene Bank Accession No.** |
| --- | --- | --- | --- |
|  |  |  |  |
| GAPDH | Forward | GAACATCATCCCAGCGTCCA | NC_006088.5 |
|  | Reverse | CGGCAGGTCAGGTCAACAAC |  |
| IL2RB | Forward | TCCAATTGATCCCAGTGCCC | NC_006088.5 |
|  | Reverse | TGGCTGTATTGGCTTGGGAG |  |
| CD8A | Forward | AGCCACGTCAACAAGGACAT | NC_006091.5 |
|  | Reverse | ACGATGGTGACGATCAGAGC |  |
| NK lysin | Forward | GATGGTTCAGCTGCGTGGGATGC | DQ186291 |
|  | Reverse | CTGCCGGAGCTTCTTCAACA |  |
| IFNG | Forward | CCTGATGGCGTGAAGAAGGT | NM_205149.1 |
|  | Reverse | CTCCTCTGAGACTGGCTCCT |  |
| Granzyme K | Forward | CGGGAAGCAACTGTTGAAAT | XM_423832 |
|  | Reverse | GAGTCTCCCTTGCAAGCATC |  |
| Granzyme A | Forward | ACTCATGTCGAGGGGATTCA | NM_204457.1 |
|  | Reverse | TGTAGACACCAGGACCACCA |  |
| Perforin | Forward | ATGGCGCAGGTGACAGTGA | XM_425355 |
|  | Reverse | TGGCCTGCACCGGTAATTC |  |
| JARID2 | Forward | CATGCACTAAGGAGGTGGGG | NC_006089.5 |
|  | Reverse | CTGGCGTACTCTTTCCAGCA |  |
| IL18RAP | Forward | TTCCGAGGGCTCTGAAACAC | NC_006088.5 |
|  | Reverse | GCAAGCTGCCTTGAGGGTAT |  |
| IRAK2 | Forward | TGAACAGATGTCTCGGGTGC | NC_006099.5 |
|  | Reverse | TGGACCATCACATGCCAGAA |  |
| ROCK1 | Forward | ACTGGGTTTTACGTTGTCGTTT | NC_006089.5 |
|  | Reverse | TACAAAGCAAGAGGCCGACA |  |

| **Target** | **Primer** | **Sequence (5’~3’)** | **Gene Bank Accession No.** |
| --- | --- | --- | --- |
|  |  |  |  |
| CCL4 | Forward | CAGACTAGAAAGGCCCACGG | NC_006106.5 |
|  | Reverse | CTCCCTTAAATGCGCTCCCT |  |
| CCL5 | Forward | GATGACTGCCGTAGCTGTGT | NC_006106.5 |
|  | Reverse | GCCCCAAACGGAGATGAAGA |  |
| FOXO3 | Forward | GTGCAGCAATGCAGTGTCAA | XM_001234495.6 |
|  | Reverse | ACACCAAAGCCTTCCCCAAA |  |
| FOXO1 | Forward | AGTTGGGTGTCAGGCTAGGA | NC_006088.5 |
|  | Reverse | TGCTGCCAACTCTGACGAAA |  |
| CCND1 | Forward | TGTCGTTCGAACCCCTCAAG | NC_006092.5 |
|  | Reverse | TTGCAGTAACTCGTCGGGTC |  |
| GATA3 | Forward | CACAGAAGGCAGGGAGTGTG | NM_001008444.1 |
|  | Reverse | GATCGCCGTTGGCATTTCTC |  |
| IL31RA | Forward | GCTGGGGCAATGTGCACTAA | NC_006127.5 |
|  | Reverse | GCCAACACAAACCTCCCTCT |  |
| FOXP3 | Forward | CCTAAAGAGCCCCAGCAACA | NM_001024827.1 |
|  | Reverse | TGGCCAGGCTGGATTGTTAG |  |
| S1PR4 | Forward | CCGAGCTCAGAATGGACCTC | NC_006115.5 |
|  | Reverse | GAGCCCGATCCTGATACAGC |  |
